# Reliability and Accuracy of Portable Devices for Measuring Countermovement Jump Height in Physically Active Adults: A Comparison of Force Platforms, Contact Mats, and Video-Based Software

**DOI:** 10.3390/life14111394

**Published:** 2024-10-29

**Authors:** Amritashish Bagchi, Shiny Raizada, Rohit K. Thapa, Valentina Stefanica, Halil İbrahim Ceylan

**Affiliations:** 1 Symbiosis School of Sports Sciences, Symbiosis International (Deemed University), Pune 412115, India; amritashish.bagchi@ssss.edu.in (A.B.); shiny.raizada@ssss.edu.in (S.R.); rohit.thapa@ssss.edu.in (R.K.T.); 2 Department of Physical Education and Sport, Faculty of Sciences, Physical Education and Informatics, National University of Science and Technology Politehnica Bucharest, 110040 Pitesti, Romania; 3 Department of Physical Education of Sports Teaching, Faculty of Sports Sciences, Atatürk University, Erzurum 25240, Türkiye

**Keywords:** plyometric exercise, athletic performance, physical fitness, validity, testing battery, motor activity

## Abstract

Measuring countermovement jump (CMJ) height accurately is essential for evaluating lower-body explosive power in athletes and other active populations. With technological advancements, various portable tools have been developed for this purpose, including force platforms, contact mats, and video-based software. This study aimed to (a) investigate the test–retest reliability of the KINVENT K-Deltas force platform for CMJ height measurement and (b) compare its accuracy with a contact mat (Chronojump, Spain) and a video-based software (My Jump app, version 3). Twenty-two physically active collegiate athletes (mean age of 19.7 ± 1.2 years) from various sports backgrounds completed five CMJ trials with simultaneous height measurements using all three tools. Intra-class correlation coefficients (ICC), Cronbach’s alpha, and coefficient of variation (CV) were calculated to assess reliability. In contrast, Pearson correlations and Bland–Altman plots were used to compare device results. The K-Deltas force platform exhibited high test–retest reliability (ICC = 0.981), closely matching the contact mat (ICC = 0.987) and the My Jump app (ICC = 0.986). Correlations between the instruments were strong (force platform vs. contact mat: r = 0.987; force platform vs. My Jump: r = 0.987; contact mat vs. My Jump: r = 0.996), with no between-instrument differences (*t*-test *p* = 0.203–0.935, effect size ≤ 0.01–0.16), demonstrating the interchangeability of these tools for practical purposes. However, Bland–Altman analysis revealed limits of agreement between the devices, indicating small but consistent measurement differences. While all instruments were reliable, discrepancies in the absolute values suggest practitioners should consider device-specific variations when comparing CMJ data. These findings highlight the reliability of the K-Deltas force platform as a viable alternative for measuring CMJ height, though differences between devices should be accounted for in applied settings. Therefore, the portable force plates can monitor training, predict injury risk, assess neuromuscular fatigue, and lead to informed decision-making.

## 1. Introduction

The countermovement jump (CMJ) assesses the stretch-shortening cycle (i.e., eccentric, amortization, and concentric phases) function of the lower body and is amongst the most frequently used tests to measure lower body strength and power capabilities due to the simplicity of the testing procedure [1]. Indeed, previous studies have reported that CMJ requires the least number of familiarization sessions compared to other vertical jump assessments [2,3,4]. Due to the test being simple to learn and perform, CMJ is used by practitioners for multiple reasons. For example, CMJ is used to evaluate the effectiveness of training [5], monitor neuromuscular fatigue [6], measure inter-limb asymmetry [7], predict injury risk [8], and guide the return to sports decision-making process [9]. Similarly, researchers have conducted studies in various sports where the CMJ test was used to assess athletes’ explosive qualities and their relationship with other variables [10,11]. Of note, CMJ has also been reported to be associated with other physical fitness outcomes such as sprinting, jumping, and change in direction [5]. In addition, the CMJ tests have been suggested to differentiate between elite versus sub-elite athletes [12,13] and professional versus amateur athletes [14,15].

With the advancement of science, several technologies (e.g., force platforms) have emerged to measure the CMJ performance of individuals, with the laboratory-based force platform considered the gold standard [16]. However, many portable technologies have emerged as we transition from laboratory-based testing (i.e., athletes visiting the laboratory) to creating a mobile laboratory (i.e., bringing the laboratory to the athlete). Some examples of such portable technologies are video-based mobile software (e.g., My Jump Lab [17]), contact platforms (e.g., Chronojump [18]), and wireless force platforms. Although new technologies are frequently developing and older technologies are becoming redundant, assessing the reliability and validity of the new technologies is important [19,20]. In simple words, the technology should provide the same measurement if conducted multiple times (i.e., reliability), and it should measure the outcome that it intends to measure (i.e., validity) [21]. Moreover, without knowing the validity and reliability of technology, practitioners would have very little confidence in the outcomes generated by the technology.

Therefore, assessing the validity and reliability of new technologies is important. For example, the video-based mobile software has been found reliable when compared against force platforms (r = 0.98, *p* ≤ 0.01, interclass correlation coefficient [ICC] = 0.997) and contact mats (r = 0.99, *p* ≤ 0.01, ICC = 0.948) [17]. Similarly, portable contact mats have also been found reliable (r = 0.99, *p* ≤ 0.01, ICC = 0.99) against a proprietary jump mat (Globus Ergo Tester) [18]. In line with new emerging technologies, portable force platforms are also gaining popularity with a significant price decrease compared to the very high cost of embedded force platforms. This makes portable force platforms accessible to a larger population. However, with the popularity of portable force platform technology, it is also very important to assess the validity and reliability of those instruments.

One of the new portable force platform technologies (K-Deltas) on the market is manufactured by Kinvent Physio (Montpellier, France). The K-Deltas measure the CMJs with a high sampling rate of 1000 Hz and have a user-friendly interface. It is equipped with a unidirectional strain gauge (vertical axis; records vertical force data) that is easily connected to a mobile device (Android or iOS) via Bluetooth and can be a good alternative for traditional in-ground force platforms [22,23]. As discussed previously, ensuring the validity and reliability of such new technologies is important in maintaining scientific integrity and enhancing the confidence of practitioners in using those technologies [24]. For instance, Mylonas, Chalitsios, and Nikodelis [23] have established the validity of the K-Deltas using the gold standard Bertec Force platform during bipedal stance, measuring the ground reaction force and center of pressure. Considering the information and studies mentioned above, CMJ is crucial for assessing lower-body explosive power, but the tools used for measurements, such as force platforms, contact mats, and video-based software, vary in accessibility and cost. While force platforms are the gold standard, they are often expensive and less portable. This study addresses the gap in the literature by comparing the test-retest reliability of the K-Deltas force platform with a contact mat and the My Jump app, aiming to determine if more affordable, portable tools can reliably measure CMJ height. By providing comparative reliability data, the study results may offer valuable insights for practitioners, potentially expanding the use of CMJ testing tools in various settings and making accurate assessments more accessible across different contexts. To the best of the author’s knowledge, no studies have analyzed the reliability of K-Deltas force platforms for measuring CMJ height. Therefore, this study aimed to assess the test-retest reliability of the portable K-Deltas force platforms to measure CMJ height. In addition, the study also aimed to compare the force platform-derived jump height to the Contact mat (Chronojump, Spain) and My Jump (with an iPad) app. It was hypothesized that K-Deltas force platforms would have high test-retest reliability in comparison with Contact Mat and My Jump app.

## 2. Materials and Methods

### 2.1. Participants

A total of twenty-two healthy physically active participants (6 females and 16 males; age: 19.68 ± 1.21 years; body mass: 63.32 ± 12.77 kg; height: 171.65 ± 9.23 cm; body mass index: 21.35 ± 2.95 kg/m^2^) volunteered (via convenience sampling) to be a part of the study. Each participant was instructed to perform five CMJ (total 110 jumps). G-Power software 3.1.9.7. (University of Dusseldorf, Dusseldorf, Germany) was used to determine the sample size (110 jumps recorded by three distinct instruments resulted in a total of 330 CMJ data points), assuming power = 0.95, alpha error < 0.05, and effect size f = 0.22. The inclusion criteria for the study required participants to be currently enrolled as students in a Sports and Exercise Science program, with no recent injury or any other medical conditions that could interfere with performance and safety. Additionally, participants required at least two years of training experience (minimum 6 h of training per week) and must have actively participated in collegiate-level sports such as football (soccer), tennis, gymnastics, volleyball, wrestling, swimming, or table tennis. Participants with a history of surgeries or severe lower extremity injuries requiring medical attention and follow-up in the last 12 months were excluded. Those currently involved in another study that might interfere with their participation were also excluded. Participants with incorrect CMJ techniques during the end of the familiarization session were excluded from the study to maintain data integrity and participant safety. The performance of CMJ was visually inspected by an accredited strength and conditioning coach and a sports scientist. The testing protocols, the study’s objective, and the potential risks and benefits of the study were explained to the participants before the study was conducted. After that, informed consent forms were signed by the participants. The study was approved by the Institute Research Committee of Symbiosis School of Sports Science, Symbiosis International (Deemed University), and conducted according to the Declaration of Helsinki’s guidelines.

### 2.2. Experimental Procedure

#### 2.2.1. Contact Mat

The Contact Mat (Chronojump Boscosystem, Barcelona, Spain) was placed and fixed over the Kinvent K-Deltas portable force platforms with adhesive tape (Supplementary Figure S1). Monitoring of the setup was performed regularly, i.e., on the completion of the fifth trial of every participant (i.e., before the trials of the next participants). To maintain consistency across trials and participants, the placement of the contact mat on K-Deltas was marked using a marker. The contact mat (size: DIN-A2 420 × 590 mm) was connected to a microcontroller using an RCB cable, and a USB cable from the same microcontroller was connected to the laptop through a USB port. An open-source software (Chronojump, version 2.3.0-79) was used to record the data on jump height from the contact mat.

#### 2.2.2. Force Platform

The KINVENT Physio software (version 2.11.2) installed on an iPad (Apple Inc., Cupertino, CA, USA) was used to record data from the portable force platform. The portable force platforms were connected via Bluetooth (BLE 5.1) to the iPad. The force platforms recorded the data with a sampling frequency of 1000 Hz.

#### 2.2.3. Video-Based Mobile Software

Simultaneously, an iPad Air (Apple Inc., Cupertino, CA, USA) device was used to record the participants’ CMJ trials in the sagittal plane. The camera was placed approximately three meters away and one meter above the ground (Supplementary Figure S2). The same camera setting was used across two weeks of data collection. The videos were recorded at a frequency of 240 frames per second. Thereafter, the CMJ performance (jump height in centimeters) was extracted from the videos via the My Jump app installed on the same iPad Air device. Two independent assessors conducted the data extraction for all the CMJ jumps. One of the assessors was not the author of this study. The calculation of jump height using the application and contact mat was conducted using protocols set in previous studies [25,26].

The experimental setup for data collection was conducted in an indoor laboratory environment, ensuring optimal lighting and adequate temperature. The force platform was positioned on a concrete surface to ensure stability and accuracy in the jump height measurement during the assessment. The data collection was completed in two weeks.

### 2.3. Data Collection

#### 2.3.1. Familiarization Sessions

One week before the start of the data collection, two familiarization sessions, 60 min each, were conducted for the participants. The session included a demonstration of technique with instruction (15 min), a standard warm-up (10 min), low- to moderate-intensity jumps with sufficient recovery, instructor feedback (30 min), and cool-down (5 min). Detailed instructions followed by demonstrations were given by an accredited strength and conditioning coach and sports scientist. The first session focused on the correct jumping and landing techniques. During the second familiarization session, minor corrections in the jump technique were made if required. Both sessions ensured the athletes’ preparedness to perform CMJ with proper technique. The final selection of participants eligible for the study was conducted during this stage (i.e., assessing the correct CMJ technique by participants).

#### 2.3.2. Warm-Up

On the testing day, the participants performed a standard (RAMP) 10 min of warm-up before the performance of CMJ. The warm-up consists of 4 min of low to moderate-intensity on-the-spot jogging with one minute of high knees, two minutes of body-weight squats, three minutes of leg swings, and walking lunges with twist and arm swings. After that, the participants were allowed to perform low-moderate effort CMJs for two minutes.

#### 2.3.3. Testing Procedure

Before the testing day, participants were instructed not to be involved in any strenuous physical activity 24 h before the testing that might affect their participation. They were asked to wear comfortable clothing to allow ease of movement and proper athletic footwear. For the testing, the participants were instructed to stand on the force platform (with the contact mat securely placed over it). The participants were asked to stand straight, stable, with their hands on their hips. Once the participant was ready, all the administrators recorded the CMJ performance for each jump using the three instruments (i.e., force platforms, contact mats, and My Jump). Participants were allowed to perform sub-maximal trials before performing the actual jumps. Each participant performed a total of five CMJ trials with hands-on-hips, with each trial separated by one minute of recovery [27]. The participants were instructed to perform CMJ with moderate to high intensity, focusing on the correct technique. This was requested to record data across the jump height spectrum (as jump intensity would not affect the study’s outcome).

### 2.4. Statistical Analysis

Descriptive statistics with mean and standard deviation values were used to characterize the central tendency and scatteredness of the dataset. The Kolmogorov–Smirnov test was used to assess the data’s normality. Intraclass Correlation Coefficient (ICC; two-way random single measures [absolute agreement]), Pearson Product Moment Correlation Coefficient, one-way ANOVA, and independent sample *t*-test were employed to analyze the three instruments’ results. Levene’s Test was used to measure the homoscedasticity among the groups wherever applicable. A Bland–Atman plot was used to study the bias between the mean difference in the two instruments and to describe an agreement between these instruments. In addition, ICC, Cronbach Alpha, and Coefficient of variation (CV) were used to analyze the test-retest reliability of the collected data. ICC values were categorized based on the lower bound of the 95% confidence interval (CI) into poor (<0.50), moderate (0.50–0.75), good (0.75–0.90), and excellent (>0.90) reliability [28]. A correlation value of r = 0.10 meant a low correlation, r = 0.30 meant a medium correlation, and r = 0.50 meant a higher correlation [29]. Effect sizes (ES) were calculated as Hedge’s *g* and were interpreted as trivial (<0.2), small (0.2–0.6), moderate (>0.6–1.2), or large (>1.2–2.0) [30]. The CV, indicating the typical error of measurements as a percentage of dispersion around the mean, was acceptable if below 10% [31]. Statistical software (SPSS version 24) was used for analysis, and the significance level was set at 5%.

## 3. Results

Table 1 shows the results of descriptive statistics, comparative analysis, ICC, and correlation values from the jump height assessment during CMJ. Individual participants’ data across the three measurement devices are presented in Supplementary Figure S3. No significant differences (*p* = 0.368) were reported between the force platform, contact mat, and My Jump app in the jump height of the participants. A nearly perfect correlation coefficient (r = 0.987; *p* < 0.001) with excellent reliability levels (ICC = 0.993) was found between the force platform and contact mat (non-significant differences *p* = 0.935, ES < 0.01 [trivial]). Similarly, there was no significant difference (*p* = 0.241, ES = 0.16 [trivial]) reported between the force platform and My Jump, with a nearly perfect correlation coefficient (r = 0.987; *p* < 0.001) and excellent reliability levels (ICC = 0.987). Additionally, an almost perfect correlation coefficient (r = 0.996; *p* < 0.001) with excellent reliability levels (ICC = 0.990) was found between the contact mat and My Jump (non-significant differences *p* = 0.203, ES = 0.16 [trivial]).

The test-retest reliability statistics are presented in Table 2. The average measure values were used for reporting ICC. All the instruments reported excellent reliability levels with high Cronbach alpha values (>0.90) and acceptable CV values (<10%) (Force platform: ICC = 0.981, α = 0.983, CV% = 7.266; Contact mat: ICC = 0.987, α = 0.988, CV% = 6.057; My Jump: ICC = 0.986, α = 0.987, CV% = 6.625).

**Figure 1:**
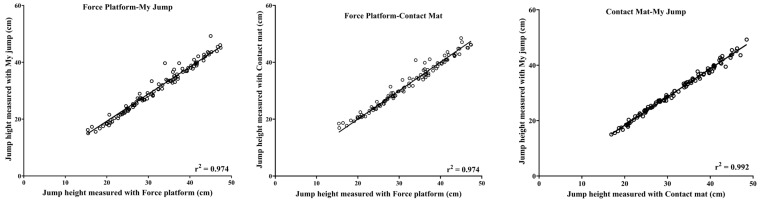
A scatter plot depicting the relationship between all the methods (i.e., force platform-contact mat, force platform-My Jump, contact mat-My Jump), along with the coefficient of determination R^2^.

**Figure 2:**
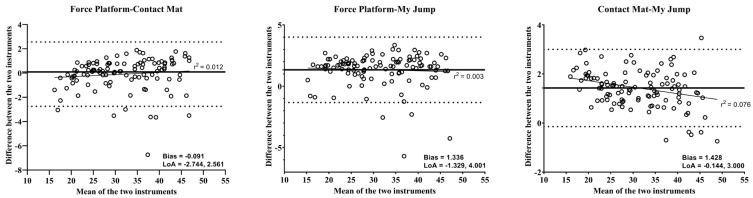
Bland–Altman Plots representing CMJ height data for all the methods (i.e., force platform-contact mat, force platform-My Jump, contact mat-My Jump). The thick central line represents the mean difference between the two methods, while the dotted lines represent ± level of agreement (±1.96 standard deviations), and the thin line represents the coefficient of determination R^2^.

The scatter plot graph displays an almost perfect correlation between the instruments in Figure 1. The Bland–Altman plot (Figure 2) depicts a large limit of agreement between the instruments (i.e., force platform vs. contact mat: LoA = −2.744, 2.561; Bias = −0.091, force platform vs. My Jump: LoA = −1.329, 4.001; Bias = 1.336, and contact mat vs. My Jump: LoA = −0.144, 3.000; Bias = 1.428).

## 4. Discussion

Reliability is an important factor that is considered while implementing the use of testing instruments. No previous study examined the reliability of portable K-Deltas. Therefore, the present study aimed to assess the test–retest reliability of the portable K-Deltas force platform to measure CMJ height and compare the force platform-derived jump height to the contact mat and My Jump app. The K-Deltas force platform was highly reliable, with an acceptable coefficient of variation acquired for both the contact mat and My Jump for measuring CMJ height. Additionally, the jump height measured from all three instruments (i.e., force platform, contact mat, and My Jump) was similar (non-significant difference; *p* > 0.05). The Bland–Atman plots also depicted a large limit of agreement between the instruments. The mean differences were relatively large but were within the 95% agreement limits (i.e., mean difference ± 1.96 SD of the difference). The spread of each score in all plots was around the bias line, and there were no cases of proportional bias [32].

Though there were no significant differences between the instruments, on average, CMJ height was found to be slightly greater in the contact mat (31.9 ± 8.3), followed by the force platform (31.9 ± 8.4), and then My Jump app (30.5 ± 8.5). Studies have reported that CMJ height is generally higher when measured with a contact mat than with a force platform [33,34,35]. Additionally, studies have also reported greater limits of agreement for CMJ height between the contact mat vs. force platform [35,36] and My Jump vs. force platform [37]. In this study, an excellent intra-class correlation coefficient with an almost perfect correlation was obtained using all three instruments (force platform vs. contact mat: ICC = 0.993, r = 0.987, force platform vs. My Jump: ICC = 0.987 r = 0.987, contact mat vs. My Jump: ICC = 0.990, r = 0.996) for measuring CMJ height. Additionally, an excellent between-trial reliability (force platform: ICC = 0.981, contact mat: ICC = 0.987, My Jump: ICC = 0.986) was reported along with an acceptable CV (7.266%, 6.057%, 6.625%).

To the author’s knowledge, this is the first study comparing the CMJ height measures of the Kinvent K-Deltas portable force platform with the Chronojump contact mat and My Jump app. Several studies were conducted previously to test the reliability and validity of the My Jump app [37,38,39] and contact mat [26,36,40] with a force platform for measuring CMJ. My Jump studies reported excellent ICC and an almost perfect correlation with the scores obtained from the force platform in CMJ. Similarly, studies on the contact mat showed good ICC [36] and a strong correlation coefficient [40] with the force platform. Similar results were obtained by Plakoutsis et al. [41], where the authors established the reliability and validity of the k-force plate with the My Jump app. The findings indicated excellent reliability (ICC = 0.999, 1.000) and validity (r = 0.999) of the K-force plate in CMJ height. The low difference (or high association) obtained between the portable K deltas and contact mat and My Jump application may be potentially due to the high frequency at which the instrument records the data. Moreover, this low difference also suggests that the portable force platforms are accurate in identifying the take-off and landing, which is required to calculate the jump height using the flight time data.

Our findings suggest that the K-Deltas portable force platform has high accuracy and could be used for field-based or laboratory-based assessments. The K-Deltas force platform is less expensive, lightweight, easy to carry, and can provide accurate feedback to practitioners. The underlying mechanisms that potentially contribute to greater neuromuscular adaptations observed during the use of the K-Deltas force platform in the countermovement jump (CMJ) test can be linked to the enhanced functionality of the stretch-shortening cycle (SSC), which is especially relevant for athletes [42]. This cycle, which is crucial in optimizing muscle performance during dynamic activities such as jumping, involves complex neuromuscular processes, including the stretch reflex and the H-reflex, as highlighted by Taube et al. [43]. These mechanisms are particularly pertinent for athletes, as they directly influence the ability to generate powerful, explosive movements.

Greater jumping performance, as recorded by the K-Deltas force platform, may indicate a more effective engagement of the SSC. This is characterized by increased muscle activation, particularly in key muscle groups such as the medial gastrocnemius, biceps femoris, and rectus femoris, which are essential for optimal performance in youth athletes [42,44]. Enhancing muscle activation contributes to the efficient storage and release of elastic energy during the eccentric and concentric phases of muscle action, respectively. This energy release is crucial for producing the forceful contractions needed to achieve higher jumps [45,46,47].

Moreover, the pre-activation of leg muscles before the jump, which is facilitated by the SSC, plays a critical role in increasing muscle stiffness. This stiffness prepares the agonist muscles to better resist the high-impact loads encountered during the vertical jump, thereby improving overall jump performance [43]. The ability of the K-Deltas portable force platform to capture these nuances in muscle activity and performance makes it an invaluable tool for coaches and physical trainers who seek to assess and enhance an athlete’s vertical jump capabilities. This device, by providing detailed insights into muscle power performance, can help tailor training programs to maximize athletic output, particularly in sports where explosive leg power is a key determinant of success.

This study has some limitations that need to be acknowledged. The participants were from different sports, including volleyball, soccer, gymnastics, tennis, table tennis, and swimming. Both male and female participants were also included, making the participants more heterogeneous. However, it did not affect the results, as the study aimed to assess the test-retest reliability of and compare the CMJ height between different instruments. Indeed, heterogeneous data represents the reliability of the instruments across a wide range of jump heights. Of note, future studies should aim to include more homogeneous data, possibly including elite athletes, to assess the sensitivity of the instrument. The test-retest reliability could have been slightly better if the participants had performed the CMJ with maximum effort (all five trials) instead of the instructed moderate- to high-intensity effort. The lack of a gold standard measure (ground-embedded force platforms) may have affected the study results. In addition, the My Jump app may have individual errors, as the data from the pre-recorded video was manually analyzed with the My Jump app by two assessors separately. However, an excellent ICC (0.997) was reported between the assessors’ data. Additionally, future studies could investigate the use of the K-Deltas platform for measuring additional performance metrics beyond CMJ height, such as rate of force development, peak power, or reactive strength index. These parameters are critical for understanding neuromuscular adaptations and performance in various dynamic movements, and their measurement could further validate the platform’s versatility. These studies could focus on sports-specific parameters, involving elite or amateur athletes to ensure a homogeneous sample. Additionally, the authors suggest assessing the reliability and validity of K-Deltas in comparison with the gold standard laboratory-based in-ground force platform for better implication. Lastly, long-term studies examining the impact of using portable force platforms like K-Deltas on injury prevention, training adaptation, and performance enhancement would provide valuable insights. These studies could track athletes over multiple seasons to determine whether regular monitoring of jump performance with portable platforms leads to improved outcomes in terms of performance gains and injury reduction.

The practical implications of this study’s findings are highly relevant for coaches and practitioners who seek efficient and accurate methods for assessing athletic performance. The portability, affordability, and reliability of the K-Deltas force platform allow it to be a practical alternative to traditional, lab-based in-ground force platforms. These features enable practitioners to conduct frequent assessments (pre-, mid-, and post-training) in field-based settings without needing expensive or immobile equipment. For coaches, this means they can more easily integrate jump performance testing into regular training sessions, offering real-time feedback to athletes. This capability can help tailor training programs to individual athletes’ needs, monitor progress, and adjust training loads to optimize performance. Additionally, by regularly tracking neuromuscular fatigue and injury risk, practitioners can reduce the likelihood of overtraining or injury, improving athlete longevity and performance. The ability to make quick and accurate assessments using the K-Deltas platform also enables coaches and trainers to implement data-driven decisions in competitive environments, where timely insights can lead to performance enhancements and strategic advantages. The platform’s ease of use, combined with its accuracy, offers a powerful tool for both routine athlete monitoring and high-performance decision-making.

## 5. Conclusions

The present study aimed to (1) assess the test-retest reliability of the portable K-Deltas force platform for measuring countermovement jump (CMJ) height and (2) compare its accuracy with a contact mat and video-based software. Both objectives were successfully met. The findings demonstrated that the K-Deltas force platform exhibited excellent test-retest reliability, comparable to the contact mat and the My Jump app, with no significant differences in CMJ height measurements across the devices. Additionally, the high correlation between the instruments confirmed the K-Deltas force platform as a reliable and valid tool for field-based assessments. These results suggest that the K-Deltas force platform is a practical alternative to traditional laboratory-based equipment, offering practitioners a cost-effective and portable solution without sacrificing accuracy, and can quickly connect to a mobile device via Bluetooth. However, future research should explore its application across different athletic populations and performance metrics, as well as its long-term impact on athlete development and injury prevention.

## Figures and Tables

**Table 1:** Validity statistics.

	Force Platform	Contact Mat	My Jump	ANOVA		Force Platform vs. Contact Mat	Force Platform vs. My Jump	Contact Mat vs. My Jump
	Mean ± Standard Deviation			*p*-Value				
CMJ (cm)	31.9 ± 8.4	31.9 ± 8.3	30.5 ± 8.5	0.368	MD ± SE	−0.091 ± 1.123	1.336 ± 1.137	1.428 ± 1.128
					ES	<0.01	0.16	0.16
					ICC (95% CI)	0.993 (0.990–0.995)	0.987 (0.903–0.996)	0.990 (0.578–0.998)
					*r*	0.987	0.987	0.996

Note: CMJ—Counter Movement Jump. ES—effect size. MD—Mean Difference. SE—Standard Error. ICC—interclass correlation coefficient. CI—confidence interval. *r*—Pearson correlation coefficient.

**Table 2:** Test–retest reliability.

	Force Platform			Contact Mat			My Jump		
	ICC (95% CI)	α	CV (%)	ICC (95% CI)	α	CV (%)	ICC (95% CI)	α	CV (%)
CMJ (cm)	0.981 (0.964–0.991)	0.983	7.266	0.987 (0.975–0.994)	0.988	6.057	0.986 (0.974–0.994)	0.987	6.625

Note: CMJ—Counter movement jump. ICC (95% CI)—Interclass correlation coefficient with 95% confidence interval. α—Cronbach’s alpha. CV—coefficient of variation.

## Data Availability

The datasets presented in this study can be found in online repositories. The names of the repository/repositories and accession number(s) can be found at: https://figshare.com/ (accessed on 21 October 2024), https://doi.org/10.6084/m9.figshare.26936401.v1 (accessed on 21 October 2024).

## Supplementary Materials

The following supporting information can be downloaded at: https://www.mdpi.com/article/10.3390/life14111394/s1, Supplementary Figure S1: Contact mat placement on the k-delta force platform with adhesive tape; Supplementary Figure S2: Measurement setup during the data collection; Supplementary Figure S3: Illustrations of individual participant data across the three instruments.
