# Supplementary material for: Reliability and Accuracy of Portable Devices for Measuring Countermovement Jump Height in Physically Active Adults: A Comparison of Force Platforms, Contact Mats, and Video-Based Software
Source: Life (Basel). 2024 Oct 29;14(11):1394. doi: 10.3390/life14111394 (PMC11595741; doi:10.3390/life14111394)
Supplement: Supplementary file 1 [file life-14-01394-s001.zip › life-3250134-supplementary.pdf]

Supplementary Figures

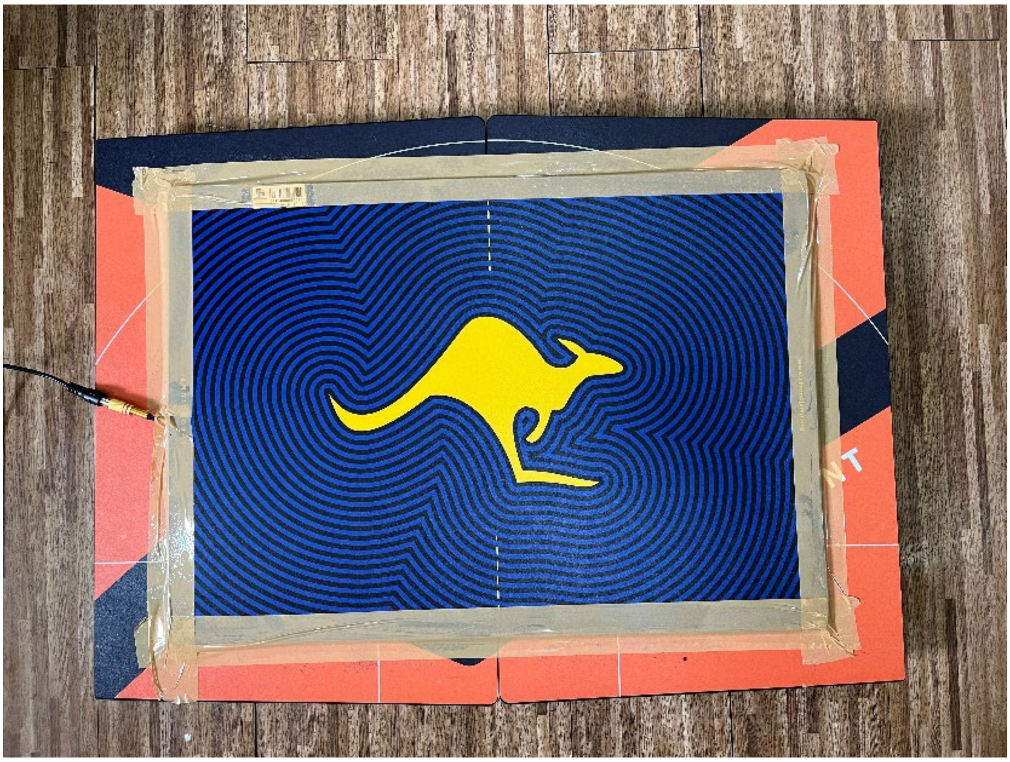

Supplementary Figure S1. Contact mat placement on the k-delta force platform with adhesive tape.

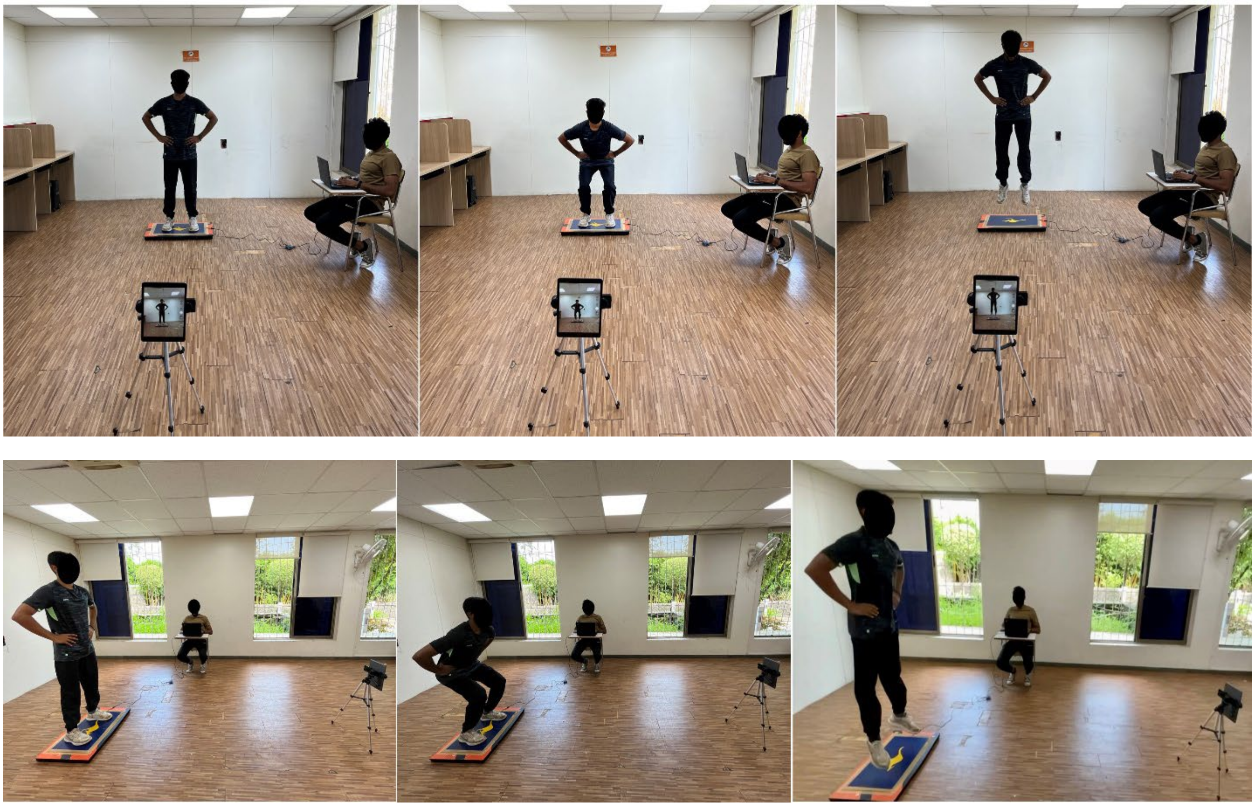

Supplementary Figure S2. Measurement setup during the data collection

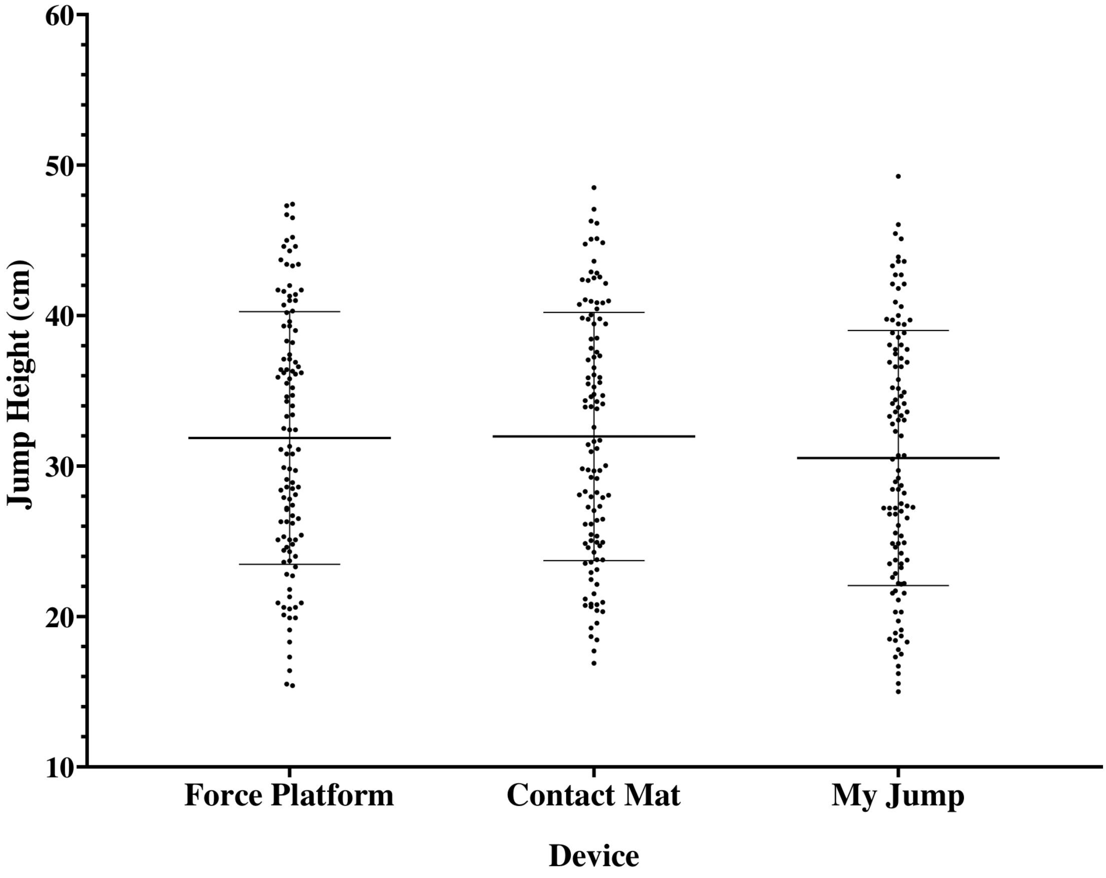

Supplementary Figure S3. Illustrations of individual participant data across the three instruments.
